# SARS‐CoV‐2 infects an upper airway model derived from induced pluripotent stem cells

**DOI:** 10.1002/stem.3422

**Published:** 2021-06-21

**Authors:** Ivo Djidrovski, Maria Georgiou, Grant L. Hughes, Edward I. Patterson, Aitor Casas‐Sanchez, Shaun H. Pennington, Giancarlo A. Biagini, Marina Moya‐Molina, Jelle van den Bor, Martine J. Smit, Git Chung, Majlinda Lako, Lyle Armstrong

**Affiliations:** ^1^ Newcells Biotech Ltd, The Biosphere Newcastle upon Tyne UK; ^2^ Biosciences Institute, Newcastle University, The International Centre for Life Newcastle upon Tyne UK; ^3^ Centre for Drugs and Diagnostics The Liverpool School of Tropical Medicine Liverpool Merseyside UK; ^4^ Vrije Universiteit Amsterdam Faculty of Science, Department of Medicinal Chemistry, Amsterdam Institute of Molecular and Life Sciences Amsterdam The Netherlands

**Keywords:** cytokines, induced pluripotent stem cells, interleukins, lung

## Abstract

As one of the primary points of entry of xenobiotic substances and infectious agents into the body, the lungs are subject to a range of dysfunctions and diseases that together account for a significant number of patient deaths. In view of this, there is an outstanding need for in vitro systems in which to assess the impact of both infectious agents and xenobiotic substances of the lungs. To address this issue, we have developed a protocol to generate airway epithelial basal‐like cells from induced pluripotent stem cells, which simplifies the manufacture of cellular models of the human upper airways. Basal‐like cells generated in this study were cultured on transwell inserts to allow formation of a confluent monolayer and then exposed to an air‐liquid interface to induce differentiation into a pseudostratified epithelial construct with a marked similarity to the upper airway epithelium in vivo. These constructs contain the component cell types required of an epithelial model system, produce mucus and functional cilia, and can support SARS‐CoV‐2 infection/replication and the secretion of cytokines in a manner similar to that of in vivo airways. This method offers a readily accessible and highly scalable protocol for the manufacture of upper airway models that could find applications in development of therapies for respiratory viral infections and the assessment of drug toxicity on the human lungs.

## Graphical abstract

We have developed a protocol to generate airway epithelial basal‐like cells from induced pluripotent stem cells, which simplifies the manufacture of cellular models of the human upper airways that are capable of supporting SARS‐CoV‐2 infection/replication and the secretion of cytokines in a manner similar to that of in vivo airways.

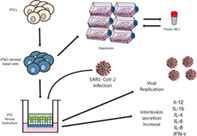

**Significance statement**

Demonstration of the ability of SARS‐CoV‐2 to infect an airway construct generated from induced pluripotent stem cells is significant since it paves the way for broader studies of viral airway infection using a system that can be manufactured reproducibly.

## 1 INTRODUCTION

The outbreak of the novel coronavirus disease, COVID‐19, caused by coronavirus SARS‐CoV‐2 has been designated as a pandemic by the World Health Organization and is currently a significant threat to human health. At the time of submission (February 2021), there were 110 958 312 million confirmed cases, of whom 2 454 901 had died.^1^ The pandemic has resulted in major challenges to global healthcare systems and has severe consequences for the global economy if the spread of the virus is not effectively controlled.

The causative agent of COVID‐19, SARS‐CoV‐2, has been shown to infect the respiratory system resulting in viral pneumonia, but it may also affect the gastrointestinal system, heart, kidney, liver, and central nervous system leading to multiple organ failure.^2^, ^3^ Previous studies have shown that SARS‐CoV predominantly infects airway and alveolar epithelial cells, and macrophages^4^ using the angiotensin‐converting enzyme 2 (ACE2) receptor for entry.^5^, ^6^ Rapid viral replication in these cells can lead to epithelial cell apoptosis causing the release of pro‐inflammatory cytokines,^7^ which can potentially cause airway damage and diminished patient survival. This is exemplified by the observation that in SARS‐CoV‐2‐infected individuals, interleukin (IL)‐6, IL‐10, and tumor necrosis factor α (TNFα) surge during illness and decline during recovery.^8^ Severely affected patients who require intensive care treatment can be distinguished by significantly higher levels of IL‐6, IL‐10, and TNFα and fewer CD4+ and CD8+ T cells.^9^ Although it is likely that the ingress of large numbers of cytokine‐secreting inflammatory macrophages into the lung tissue accounts for a significant proportion of the cytokines detected in such cases, the initial damage to the airway epithelial cells probably contributes not only to the overall concentration of cytokines but may also be responsible for recruitment of inflammatory macrophages.

Other mechanisms besides apoptosis can lead to activation of the inflammasome. The binding of SARS‐CoV‐2 to the Toll‐like receptor causes the release of pro‐IL‐1β, which is cleaved by caspase‐1, followed by inflammasome activation and production of active mature IL‐1β that mediate lung inflammation, fever, and fibrosis.^10^ To underline this, suppression of pro‐inflammatory IL‐6 has been shown to have a therapeutic effect in COVID‐19.^11^ IL‐1β can also enhance the constitutive detachment (or shedding) of an enzymatically active ectodomain fragment of ACE2 from the airway epithelial cells, an event associated with acute lung injury.^12^, ^13^ Interestingly, SARS‐CoV‐2 infection is associated with ACE2 downregulation and ectodomain shedding thought to be induced by the SARS‐CoV‐2 Spike (S) protein.^14^, ^15^ How the released form of ACE2 (the so‐called soluble or sACE2) causes lung damage is not completely clear but it seems to be tightly coupled to TNFα production, so it may be involved in inflammatory response to SARS‐CoV‐2 infection.^16^

Despite this understanding of the mechanisms by which SARS‐CoV‐type viruses damage the airway epithelia, there are no effective treatments for the resulting COVID‐19 disease. Current management of COVID‐19 is supportive, and respiratory failure from acute respiratory distress syndrome is the leading cause of mortality. In view of this, there is an urgent and currently unmet need for model systems that can function as high‐throughput preclinical tools for the development of novel, effective therapies for COVID‐19.

The use of in vitro models mimicking the human airways generated from primary pulmonary epithelial cells grown at an air‐liquid interface (ALI) has increased in popularity over the recent years. The cells can form a pseudostratified airway epithelium composed of all in vivo relevant cell types found in the airway epithelium including rare types such as pulmonary neuroendocrine cells and ionocytes.^17^ This type of model has proven to be particularly useful for toxicological assessment of aerosol particles,^18^, ^19^ drug discovery,^20^ and more recently with the COVID‐19 outbreak for viral infection studies.^21^ Although these models offer significant advantages, their availability is limited to primary samples, which can significantly differ depending on the donor's genetic background and thus affecting the generated airway.

Induced pluripotent stem cells (iPSCs) offer the potential to complement the limitations of primary cells by generating a large supply of cells with the genetic background of the donor. The pathways involved in differentiation into proximal and distal airway lineages have already been established by previous groups, who successfully differentiated functional ciliated cells, mucus‐producing cells, and alveolar cells.^22^, ^23^, ^24^, ^25^, ^26^ The current methods of lung differentiation use 3D self‐forming spheroids or 2D cultures of mixed population of cells, which are more challenging when performing experiments similar to the primary airway ALI models.^27^, ^28^, ^29^ In this study, we isolated a population of basal‐like cells from differentiating iPSCs and used these to generate airway epithelial equivalents by ALI culture. We show that these comprise the cell types found in the human upper airway epithelium including functional ciliated cells, are capable of secreting mucus, and are readily infected by SARS‐CoV‐2 as demonstrated by the replication within the cells of the airway construct, release of virions into the supernatant growth media, and the presence of SARS‐CoV‐2 spike protein in specific cells. Infected constructs also secrete cytokines at levels corresponding to the behavior of the airway epithelial in vivo following SARS‐CoV‐2 infection.

## 2 MATERIALS AND METHODS

### 2.1 Generation of airway epithelial constructs using airway basal‐like cells derived from iPSCs

iPSCs lines SB‐AD2^30^ and SB‐AD3^31^ were cultured at 37°C with 5% CO_2_ on six‐well plates coated with Matrigel (BD, 354230) in mTeSR1 (STEMCELL Technologies, 85850) with daily media replacement. At 80% confluency, the cells were passaged with Versene ethylene diamine tetra‐acetic acid (EDTA) 0.02% (Lonza, BE17‐711E) for 5 minutes and transferred at a split ratio of 1:3 into fresh Matrigel‐coated plates. The cells were passaged at least twice before initiating differentiation. All iPSC cultures were performed in a class II biosafety cabinet laminar airflow tissue culture hood under a dissection microscope.

Differentiation into airway basal cells involved transit through definitive endoderm and anterior foregut endoderm stages as follows. Briefly, 90% confluent iPSCs were washed with phosphate‐buffered saline (PBS; 2 × 2 mL per well of a six‐well plate), then cultured in Advanced RPMI1640 medium (Thermo Fisher Scientific, Paisley, UK, 12633012) containing 0.02% B27 supplement (Life Technologies Paisley, UK, 17504044) supplemented with 50 U/mL penicillin/streptomycin (Thermo Fisher Scientific, 15140122), 100 ng/mL human activin A (R&D Systems, Abingdon, UK, 338‐AC), 1 μM CHIR99021 (Sigma‐Aldrich, Gillingham, UK, SML1046) and 10 μM of Y‐27632 (Chemdea Eidgewood, New Jersey, CD0141). The medium was refreshed daily for 6 days, and the cells were kept in an incubator at 37°C containing 5% CO_2_ and 95% humidity. On day 6, the medium was changed to Advanced RPMI1640 medium containing 0.02% B27 supplement supplemented with 100 ng/mL human recombinant noggin (R&D Systems, 6057‐NG) and 10 μM of SB‐431542 (R&D Systems, 1614). The medium was changed daily for 4 days. On day 10, the medium was changed to Advanced RPMI1640 medium (Thermo Fisher Scientific, 12633012) containing 0.02% B27 supplemented with 100 ng/mL of human recombinant BMP4 (Peprotech London, UK, 120‐05ET), 0.5 μM of all‐trans retinoic acid (ATRA) (Sigma‐Aldrich, R2625) and 3 μM of CHIR99021. The medium was changed every other day for 4 days.

To isolate basal airway‐like cells, the day 14 differentiated cells were washed with PBS and enzymatically detached with trypsin for 5 minutes. The detached cells were centrifuged at 300*g* and resuspended in BEGM medium (Lonza, Castleford, UK, CC‐4175) supplemented with 10 μM of Y‐27632. They were plated at a ratio of one well into six on mitotically inactivated 3T3 cells. The medium was changed every other day, until 90% confluency of basal cells is reached. The basal cells could be passaged at a ratio of 7000 cells/cm^2^ on irradiated 3T3s for at least eight passages. The cells could also be frozen at 1 million per vial in 50% BEGM, 40% fetal bovine serum (FBS), and 10% Dimethylsulfoxide (DMSO) and stored in liquid nitrogen for later use.

To differentiate these into a pseudostratified airway epithelium, basal cells at 90% confluency on mitotically inactivated 3T3 feeders were harvested as a single cell population by trypsinization, then seeded at a density of 150 000 cells per well onto the apical face of 24‐well plate cell culture inserts (ThinCerts, Greiner bio‐one, Gloucestershire, UK, 662610) with a transparent membrane (PET), with a pore diameter of 0.4 μm, precoated with Matrigel (1:100) and fibronectin (1:100) (Sigma Aldrich, F1141). The adherent cells were fed for 3 days apically and basolaterally with BEGM medium until they formed a confluent monolayer. Once confluent, the apical medium was removed, and the cells were fed PneumaCult (STEMCELL Technologies, Cambridge, UK, 05001) supplemented with heparin, hydrocortisone and Pen/Strep from the basal chamber. The cells were cultured over 4 weeks to achieve maturity and fed every other day from the basal chamber.

Mature ALI cultures prepared in this manner were fixed directly on the membrane with 4% paraformaldehyde (PFA) for 10 minutes at 3°C and then washed with PBS (3 × 1.0 mL). The tissues were then removed together with the membrane, placed into molds, and embedded in optical coherence tomography (OCT) matrix (Cell Path, Newtown, UK, KMA‐0100‐00A). The molds were placed at −20°C to solidify. Once solid, they were sectioned into 5‐ to 10‐μm slices on slides using a cryostat. The sectioned membrane was removed with PBS washes, and the slides were then stained using the same procedure as the basal cells using the same antibodies with the addition of antibodies (Tables 1 and 2). Once the staining was finalized, a few drops of Vectashield medium containing Hoechst were added to the slides then they were covered with coverslips, sealed with nail polish, and left to dry at 4°C.

**TABLE 1:** List of antibodies used for immunofluorescence and flow cytometric analysis of basal cells

Antibody name	Dilution	Species	Reference	Supplier
Integrin alpha 6	1:100	Mouse	Ab30497	Abcam, Cambridge, UK
NGFR	1:100	Mouse	12152170	Thermo Fischer Scientific
Cytokeratin 14	1:100	Mouse	Ab210414	Abcam
ΔNp63	1:400	Rabbit	ab167612	Abcam
α‐Mouse‐AlexaFluor‐488	1:1000	Goat	A11001	Invitrogen, Paisley, UK
α‐Rabbit‐AlexaFlour‐647	1:1000	Goat	A21245	Invitrogen

**TABLE 2:** List of antibodies used for immunofluorescence of air‐liquid interface cultures

Antibody name	Dilution	Species	Reference	Supplier
ΔNp63	1:400	Rabbit	ab167612	Abcam
Mucin1	1:100	Mouse	11548812	Thermo Fisher Scientific
CC10	1:100	Mouse	sc‐365992	Santa Cruz, Heidelberg, Germany
Acetylated Tubulin	1:100	Mouse	T6793	Sigma‐Aldrich
ZO‐1	1:300	Rabbit	61‐7300	Invitrogen
Synaptophysin	1:200	Rabbit	YE269	Abcam
TMPRSS2	1:900	Rabbit	ab92323	Abcam
ACE2	1:200	Goat	AF933	R&D Systems
Spike (SARS‐CoV‐2)	1:100	Rabbit	703959	Invitrogen
Goat α‐mouse‐AlexaFluor‐488	1:1000	Goat	A11001	Invitrogen
Goat Α‐rabbit‐AlexaFluor‐647	1:1000	Goat	A21245	Invitrogen
Donkey Α‐Goat‐Alexa Fluor	1:1000	Donkey	A21447	Invitrogen
Donkey α‐rabbit‐AlexaFluor‐546	1:1000	Donkey	A10040	Invitrogen
Donkey α‐mouse‐AlexaFluor‐488	1:1000	Donkey	A21202	Invitrogen

### 2.2 Characterization of basal epithelial cells by immunofluorescence and flow cytometry

Basal airway‐like cells generated in this manner were characterized by a combination of flow cytometry (Fortessa flow cytometer and FloJo analysis) and immunofluorescence (IF) (see Table 1 for a list of antibodies used). At least 10 000 cells were analyzed for each basal‐like cell sample. For IF analysis, basal‐like cells were grown on feeder layers of mitotically inactive 3T3 cells on glass coverslips and cultured in 24‐well plates. The 24‐well plates containing coverslips were washed with PBS (Thermo Fisher Scientific, 10010056) (2 × 1.0 mL) and fixed with 4% PFA (Sigma‐Aldrich, 158127) in PBS for 10 minutes at 37°C. The cells were washed with PBS (2 × 1.0 mL) then permeabilized with 1.0 mL of PBS‐0.25% Triton X‐100 for 30 minutes. The permeabilization solution was replaced with blocking solution (2% bovine serum albumin [BSA] in PBS [w/v]) followed by incubation for 1 hour. Each primary antibody was diluted in 150 μL of the blocking solution according to the concentrations shown in Table 2. The cells were then treated with the primary antibodies and incubated at 4°C (12 hours). Following this, the cells were washed with PBS (3 × 1.0 mL). Secondary antibodies (Table 2) were prepared in blocking solution (1:1000) and added on the samples for 1 hour at room temperature in the dark. The cells were washed again with PBS (3 × 1.0 mL). The coverslips were removed from the plate once the staining was finished and placed on a superfrost slide with a few drops of Vectashield medium containing Hoechst (1:10 000 as a nuclear counterstain). Coverslips were sealed with nail polish and left to dry in a dark box before storage at 4°C followed by fluorescence microscopy.

### 2.3 Hematoxylin and eosin staining

ALI cultures were fixed by incubation with 4% PFA/PBS (w/v) for 10 minutes at room temperature. The membrane containing tissues were surgically removed from the insert and sandwiched between Shandon sponges (Thermo Fisher Scientific, 85‐43) and 3‐mm Whatman paper (GE Healthcare, 9.950371) in tissue embedding cassettes. Subsequently, paraffination was performed using the Excelsior AS Tissue processor (Thermo Fisher Scientific, A82300001), and the paraffinized tissues were placed into molds. Once the paraffin had solidified, 3‐μm sections were made using a microtome. The slides were rehydrated using xylene and an ethanol series (100%, 96%, and 70%). Subsequently, the slides were stained using Mayer's hematoxylin (Sigma‐Aldrich, MHS32) and alcoholic eosin Y (Sigma‐Aldrich, HT110116) followed by dehydration using an ethanol series (80%, 96%, and 100%). Xylene‐washed slides were mechanically covered using coverslips and dried at room temperature. Histology was assessed using an AxioVert 25 inverted microscope (Zeiss).

### 2.4 Quantification of cilia beat frequency

Prior to imaging, the apical surface of the ALI cultures was washed using medium from the basal chamber. Subsequently, high‐speed videos were captured using the Nikon Eclipse Ti2 LIPSI high content imaging microscope equipped with a phase 1 phase contrast ring and a CFI S Plan Fluor LWD ×20 objective. The middle of the Prime BSI sCMOS camera was used to capture 550 images (512 × 512 pixels) over 5.5 seconds at a rate of 100 frames per second. During imaging, the atmosphere was constantly kept at 37°C, 5% CO_2_, and 95% humidity. At least three fields containing cilia for three inserts were imaged.

For analysis MATLAB was used to calculate cilia beat frequency. Here, the intensity‐time trace of each pixel was filtered for frequencies between 5 and 25 Hz using a band‐pass filter. Subsequently, the power spectrum density was calculated. The frequency of each pixel is defined by the highest peak of the power spectrum density. The ciliated cells were visualized by plotting the frequency of each pixel into a heat map. The frequency distribution of the ciliated cells was visualized by plotting the amount of nonzero pixels into a histogram. The average frequency of all nonzero pixels was compared using a student's *t* test.

### 2.5 Infection of airway epithelial constructs with SARS‐CoV‐2

To generate sufficient viral particles for infection experiments, SARS‐CoV‐2 isolate REMRQ0001/Human/2020/Liverpool was cultured from a clinical sample and passaged four times in Vero E6 cells (C1008; African green monkey kidney cells obtained from Public Health England) cultured in Dulbecco's minimal essential medium (DMEM) containing 10% FBS and 0.05 mg/mL gentamycin at 37°C with 5% CO_2_. The fourth passage of virus was cultured in Vero E6 cells with DMEM containing 4% FBS and 0.05 mg/mL gentamycin at 37°C with 5% CO_2_ and was harvested 48 hours postinoculation. Virus stocks were stored at −80°C.

Virus quantification was performed by standard plaque assays on Vero E6 cells plated at a density of 6 × 10^5^ cells per well of a six‐well plate. Aliquots of 100 μL of virus stocks over several dilutions were added to each well of a six‐well plate, covered with overlay medium then incubated at 37°C/5% CO_2_ for 72 hours, fixed with 10% formalin and stained with 0.05% crystal violet solution. The number of noncolored plaques counted in the crystal violet‐stained plate indicated the number of plaque‐forming units (PFU) of the viral dilution.

Based upon the PFU count of the virus stock, dilutions were prepared to infect airway epithelial constructs at a multiplicity of infection (MOI) of 0.01 in DMEM with 4% FBS and gentamycin (Sigma‐Aldrich, 345814‐M). Hundred microliters of virus dilution were added to the apical face of each airway epithelial construct on cell culture inserts followed by the addition of 250 μL of DMEM (Thermo Fisher Scientific, 31966047) with 4% FBS and gentamycin. At each time point, the apical surface of the construct was washed by adding 1.5 mL of PBS. This is a necessary step since the constructs survive at an ALI meaning that there is no supernatant liquid above the apical face. The added PBS “supernatant” from each infected and control construct was transferred to a 1.5‐mL microcentrifuge tube, then centrifuged (2500 rpm, 5 minutes) and stored at −80°C until needed for viral quantification. Samples of basolateral medium were collected in parallel to the PBS “supernatant” washes and to these, Triton X‐100 was added to a final concentration of 0.5% followed by incubation (room temperature, 30 minutes) to inactivate SARS‐CoV‐2. The inactivated basolateral media samples were stored in liquid nitrogen until needed for the quantification of cytokines. The cellular component of constructs at each time point was collected for the quantification of viral particles present within cells and additional constructs for each time point were fixed with 4% PFA for 30 minutes to provide infected ALI constructs for analysis by IF. Mock controls, which were treated the same way except without virus, were included alongside SARS‐CoV‐2‐infected ALI constructs.

Quantification of viral particles present in the “supernatant” PBS washes and cellular mass was determined for each time point using the Vero E6 plaque assay method. The infected constructs were fixed directly on the membrane with 4% PFA for 10 minutes at 37°C and then washed three times with PBS for 5 minutes. The tissues were then removed together with the membrane and placed into molds and covered with OCT (Cell Path). The molds were placed at −20°C to solidify. Once solid, they were cryosectioned into 10‐μm slices on slides using a cryostat. The sectioned membrane was removed with PBS washes and the slides were then stained using the same procedure as the basal‐like cells.

ALI cultures analyzed by confocal microscopy were processed as follows: 24 hours after infection, mock and SARS‐CoV‐2‐infected whole constructs were fixed in 4% PFA for 45 minutes at room temperature and washed in PBS. The constructs were then permeabilized in 0.5% Triton X‐100 in PBS for 25 minutes at room temperature, washed in PBS, and blocked in 5% BSA, 20% FBS in PBS for 1 hour at room temperature. ALI constructs were then incubated with a cocktail of primary antibodies in blocking solution at 4°C overnight (goat anti‐ACE2 1:200; rabbit anti‐Transmembrane protease, serine 2 (TMPRSS2) 1:2000, mouse anti‐Spike 1:100; Abcam). After washing, they were incubated with secondary antibodies in blocking solution containing 1 mg/mL of 4′,6‐diamidino‐2‐phenylindole for 1 hour at room temperature (anti‐goat AF488, anti‐rabbit AF555, anti‐mouse AF647; all Thermo Fisher Scientific 1:1000) and were washed further. Organoids were embedded in 1% low‐melting agarose at 30°C containing Slowfade diamond mounting oil and imaged using a Zeiss LSM880 confocal laser scanning microscope. At ×400, sections were 3D reconstructed from a series of *z*‐stacks (20‐40‐mm slices) with automatic optimal thickness and 1 Airyscan unit pinhole. Orthogonal views were generated using Zeiss Zen 3.3 software.

### 2.6 Quantification of cytokine release after infection with SARS‐CoV‐2

Quantification of cytokines was performed using the V‐PLEX Plus Viral panel 3 Human Kit purchased from Mesoscale Discovery (MSD, K15347G‐1). The lyophilized cocktail mix calibrators for Proinflammatory panel 1, Chemokine panels 1 and 4 calibrators for U‐PLEX Biomarker group 1 (calibrators 1, 3, 6, 9) were reconstituted in provided assay diluents, respectively. U‐PLEX plates were coated with supplied linkers and biotinylated capture antibodies according to the manufacturer's instructions. Proinflammatory cytokines and chemokines in basal media collected at 24, 48, and 72 hours after stimulation were detected with precoated V‐PLEX. The assays were performed according to the manufacturer's protocol at 2 hours incubation of the diluted samples and standards at 4°C. The electrochemiluminescence signal was detected by MESO QuickPlex SQ 120 plate reader (MSD) and analyzed with Discovery Workbench Software (v4.0, MSD). The concentration of each sample was calculated based on the four‐parameter logistic fitting model generated with the standards (concentration was determined according to the certificate of analysis provided by MSD). The graphs were plotted with GraphPad and *t* test was performed for statistical significance, *P value >.05, **P value >.001.

## 3 RESULTS

### 3.1 iPSCs‐derived airway basal cells generate airway epithelial constructs at an ALI

**FIGURE 1:**
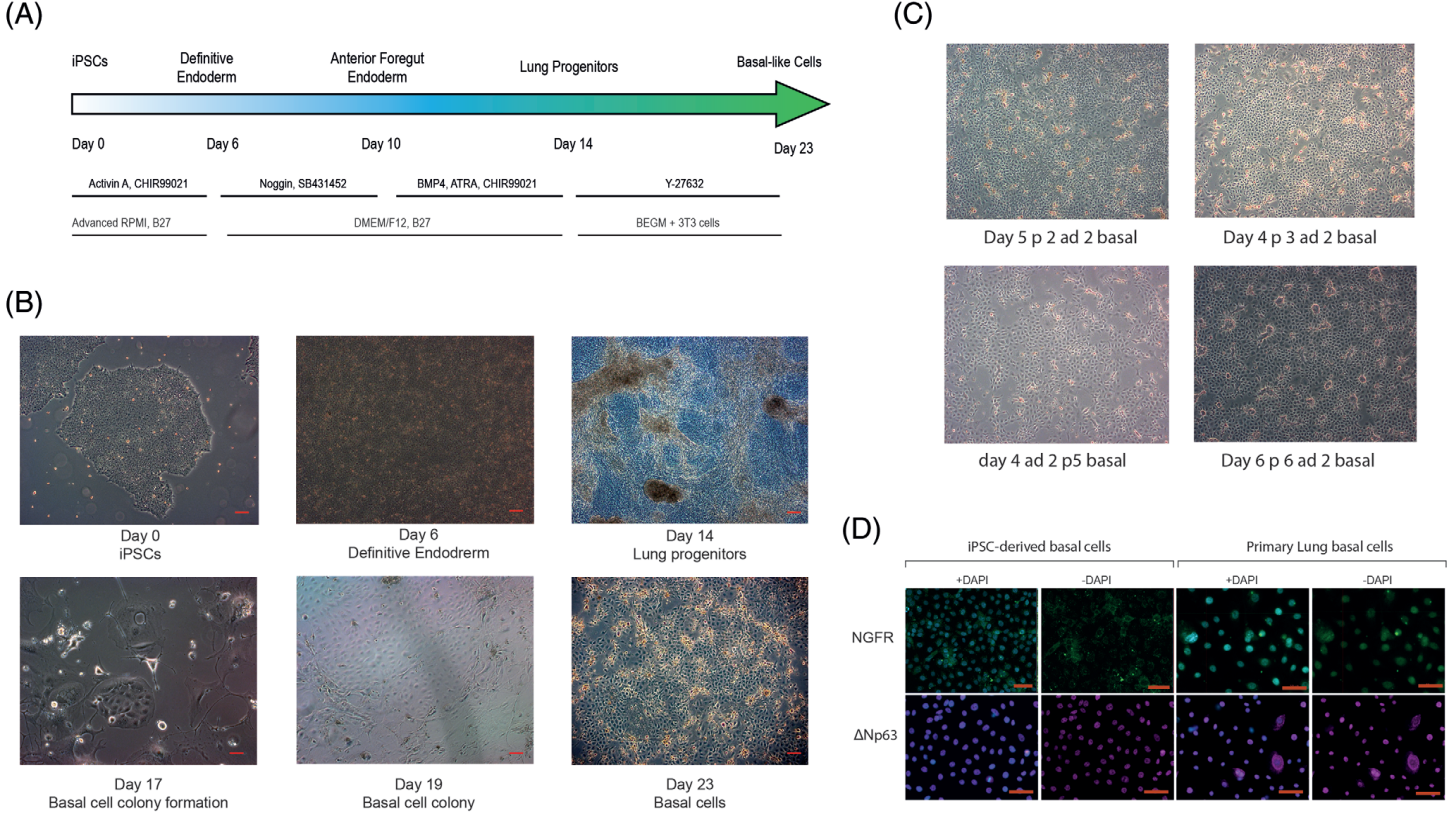
Generation of induced pluripotent stem cell (iPSC)‐derived airway basal‐like cells. A, The schedule of growth factor additions and media composition for directed differentiation of iPSC via definitive and anterior foregut endoderm stages. After day 14, cells were cultured at low density on mitotically inactivated mouse 3T3‐J2 feeders, B, in the presence of primary airway medium and Rho kinase inhibitor (Y27632) to generate colonies of basal cells that expressed markers characteristic of airway basal cells derived ex vivo, C, and were expandable for several passages, D. Scale bars (red) in B, C, and D represent 50 μm. Representative images from six experiments in both iPSC lines are shown

iPSCs were directed to differentiate to definitive endoderm by adding Activin A and CHIR99021 for 6 days according to the protocol described by Konishi et al.^25^ Anterior foregut endoderm was subsequently induced by dual inhibition of the Transforming growth factor‐beta (TGF‐beta) signaling by Noggin and SB431542 for 4 days and then inhibition of the Wnt Pathway by CHIR, ATRA, and BMP4 for additional 4 days was used to generate a mixed population of lung progenitors (Figure 1A). These cells were cultured at low density on mitotically inactivated mouse 3T3‐J2 feeders in the presence of primary lung airway medium BEGM (Lonza, CC‐4175 containing the Rho‐kinase inhibitor Y27632). Colonies of epithelial‐like cells grew within 10 days of plating (Figure 1B), and these expressed markers previously identified for airway basal cells markers keratin 14 (KRT14), integrin alpha 6, NGFR, and ΔNp63^32^, ^33^, ^34^ detectable by IF (Figures 1C and S1) and/or quantifiable by flow cytometry (Figure S1A). To our knowledge, this method of enriching and expanding airway basal‐like cells derived from iPSCs has not been reported previously. These cells responded well to cryopreservation in FBS plus 10% DMSO and recovered post‐thaw to establish colonies on the 3T3‐J2 feeders with BEGM supplemented with Rho‐kinase inhibitor, Y27632 (Figures 1D and S2). In contrast, iPSC‐derived basal‐like cells cultured in BEGM supplemented with Rho‐kinase inhibitor, Y27632 but in the absence of feeder cells, could not be maintained past passage 4, indicating that culture on 3T3‐J2 mitotically inactivated cells is critical for their continued expansion. Gene expression studies indicated an upregulation of basal epithelial cell markers *KRT6A*, *KRT5*, and *KRT17* upon expansion on 3T3‐J2 mitotically inactivated feeder cells (Figure S3, *P* < .001 for all markers).

**FIGURE 2:**
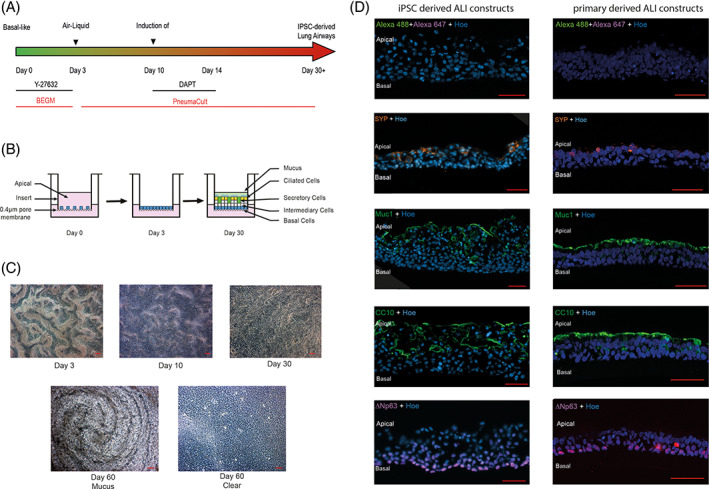
Generation of airway epithelial constructs from basal‐like cells. A, To induce airway differentiation, induced pluripotent stem cells (iPSCs)‐derived basal cells were plated on a transwell insert, left to grow to confluency then fed from the basal side leaving the apical side in contact with the air, B. C, Cells with beating cilia were visible as early as 2 weeks of culture and a mucus layer was present on the apical surface of the construct with multiple hole‐like structures. D, Left‐hand side panel: Sections of constructs indicate the presence of a pseudostratified epithelium, in which cells expressing the basal cell marker, delta Np63 are enriched in a layer adjacent to the membrane of the transwell insert. Differentiation of the basal cells into the other cell types present in the pseudostratified epithelium is indicated by the presence of club cell protein 10 (CC10) (club cells), and expression of mucin‐1 (Muc1) (goblet cells). Expression of synaptophysin (SYN), D, suggests the presence of pulmonary neuroendocrine cells. The same immunofluorescence analyses were performed in primary lung air‐liquid interface (ALI) constructs and shown in the right‐hand panel. Cells are co‐stained with Hoechst 33342 (Hoe) to indicate positions of nuclei. Scale bars in C and D represent 50 μm

To induce airway differentiation, iPSCs‐derived basal cells were allowed to grow to confluency on the 3 T3‐J2 feeder layers then harvested as a single‐cell population by trypsinization after removal of the bulk of the feeder cells by exposure to 0.48 mM sodium EDTA (Versene, Gibco). The single cells were plated on transwell inserts (ThinCerts, Greiner bio‐one, 662610) (see Figure 2A), left to grow to confluency (day 3) and then they were fed from the basal side leaving the apical side in contact with the air (see Figure 2B). To differentiate the cells into airway epithelia, the commercial PneumaCult media was used with the addition of 10 μM N‐[N‐(3,5‐Difluorophenacetyl)‐L‐alanyl]‐S‐phenylglycine t‐butyl ester (DAPT) between differentiation days 10 and 14. Cells with beating cilia were visible as early as 2 weeks of culture (Figure 2C) and a mucus layer was present on the apical surface of the construct with multiple hole‐like structures (Figure 2C). IF analysis indicated the presence of a pseudostratified epithelium (Figure 2D), in which cells expressing the basal cell marker, p63 were enriched in the basal layer adjacent to the membrane of the transwell insert. Differentiation of the basal cells into the other cell types present in the pseudostratified epithelium was indicated by the presence of club cell protein 10 (club cells, Figure 2D) and the expression of mucin‐1 (goblet cells, Figure 2D). The presence of putative pulmonary neuroendocrine cells is indicated by the expression of synaptophysin (Figure 2D). The expression of all the four markers was similar between iPSC‐derived and primary lung cell‐derived ALI constructs (Figure 2D), although for MUC1 and CC10, the expression in the primary lung cell‐derived ALI constructs was stronger and localized in the very superficial layer.

**FIGURE 3:**
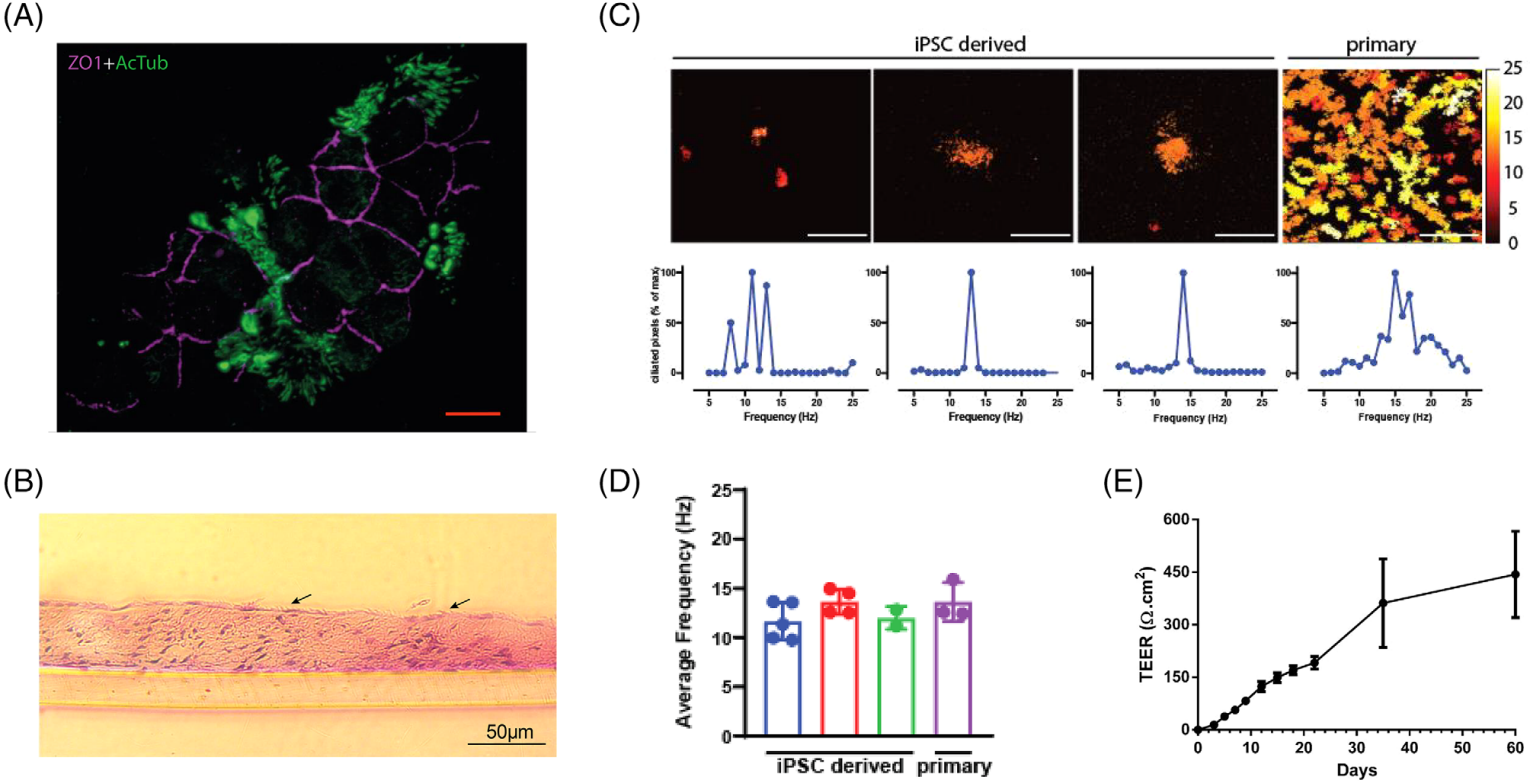
Airway epithelial constructs possess tight junctions and functional cilia. A, The apical surface of the pseudostratified epithelium comprises ciliated epithelial cells capable of forming tight junction indicated by the presence of ZO‐1 and cilia indicated by the presence of acetylated tubulin (AcTub). Scale bars represent 10 μm. B, Cilia are also visible in hematoxylin and eosin‐stained sections (indicated by arrows) as early as 2 weeks and the functionality of these structures is evident from their motility quantified in our measurements of ciliary beat frequency C,D. E, The high levels of trans‐epithelial electrical resistance (TEER) values support the establishment of an epithelial barrier. Data is shown as mean ± SEM, n = 3

The apical surface of the pseudostratified epithelium comprises ciliated epithelial cells capable of forming tight junction indicated by the presence of ZO‐1 and cilia indicated by the presence of acetylated tubulin (Figure 3A). Cilia are also visible in hematoxylinand eosin‐stained sections (Figure 3B), and the functionality of these structures is evident from their motility quantified in our measurements of ciliary beat frequency (Figure 3C). Pseudostratified airway epithelia constructs generated from iPSCs‐derived basal cells comprised patches of ciliated cells when compared with higher density of ciliated cells in similar constructs made from ex vivo‐derived primary airway basal cells. Despite this, the beat frequency of cilia presents on the iPSCs‐derived constructs was in a similar range to those derived from primary basal cells (12.4 ± 1.6 and 13.6 ± 2.0 Hz, respectively, see Figure 3D). Moreover, the ability to form tight junctions between the ciliated epithelial cells probably contributed to the trans epithelial electrical resistance (TEER) values in the range of 250 to 550 Ω/cm^2^ by day 60 (Figure 3E) of culture indicating establishment of an epithelial barrier.

### 3.2 Airway epithelial constructs generated from iPSCs‐derived basal cells are permissive for SARS‐CoV‐2 infection and replication

**FIGURE 4:**
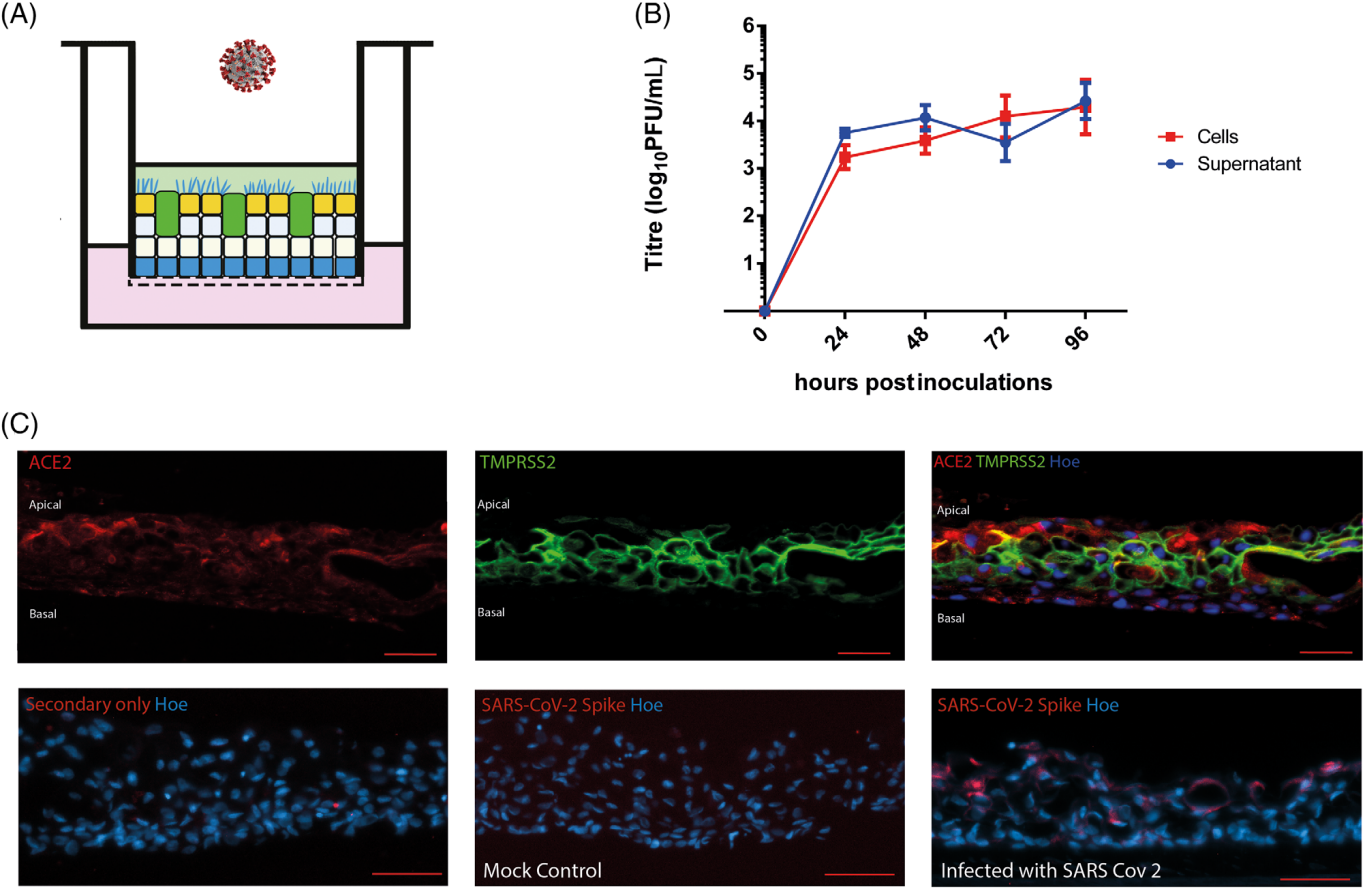
Response of airway epithelial constructs to infection by SARS‐CoV‐2. A, Schematic presentation of SARS‐CoV‐2 infection showing addition of virus on the apical face of induced pluripotent stem cells (iPSCs)‐derived lung air‐liquid interface (ALI) constructs. B, Following inoculation of the virus onto the apical face of the construct at a multiplicity of infection (MOI) of 0.01, the number of viral particles present in the cells of the construct and in the PBS “supernatant” washes collected from the apical ALI was assessed with the plaque unit forming assay. Data shown as mean ± SD, n = 3. Zero hours indicate the plaque forming unit ability of supernatant or cell lysate taken just before SARS‐CoV‐2 infections. C, Lung airway epithelial constructs generated in this study express both (angiotensin‐converting enzyme 2 [ACE2] and TMPRSS2) proteins required for SARS‐CoV‐2 invasion. IF localization of the SARS‐CoV‐2 spike protein showing that infected cells are clearly visible in greater numbers of the apical face of the construct. All scale bars (red) represent 50 μm

The SARS‐CoV‐2 genome encodes several structural proteins including the glycosylated spike protein (S‐protein) that mediates cell invasion by binding to ACE2 on the surface membrane of target cells.^35^, ^36^, ^37^ Cell invasion also requires S‐protein priming, which is facilitated by the host cell serine protease TMPRSS2. Airway epithelial constructs generated in this study co‐expressed both proteins required for SARS‐CoV‐2 invasion at the apical face (Figures 4C and S4). We exposed the iPSC‐derived ALI constructs to SARS‐CoV‐2 at a MOI of 0.01 on the apical face (Figure 4A). Cell and supernatant samples were harvested at multiple time points after infection and processed for plaque‐forming unit assays as shown in Figure 4B. SARS‐CoV‐2 productively infected the iPSC‐derived ALI lung constructs, as assessed by live virus titrations on VeroE6 cells (Figure 4B). SARS‐CoV‐2 titers remained stable at 72 hours after infection and increased significantly in the supernatant from 72 to 96 hours (*P* = .05). Culture supernatants contained similar levels of infectious virus compared with lysed cellular constructs, suggesting that the virus was secreted apically. These data underline the ability of iPSC‐derived ALI lung constructs not only to permit entry of viral particles from the initial inoculum but also to replicate new virions and release them from the cells. IF analysis of sections and whole ALI lung constructs showed the presence of Spike protein in ACE2^+^TMPRSS2^+^ cells at the apical face (Figures 4C and S4), further confirming their infection with SARS‐CoV‐2.

**FIGURE 5:**
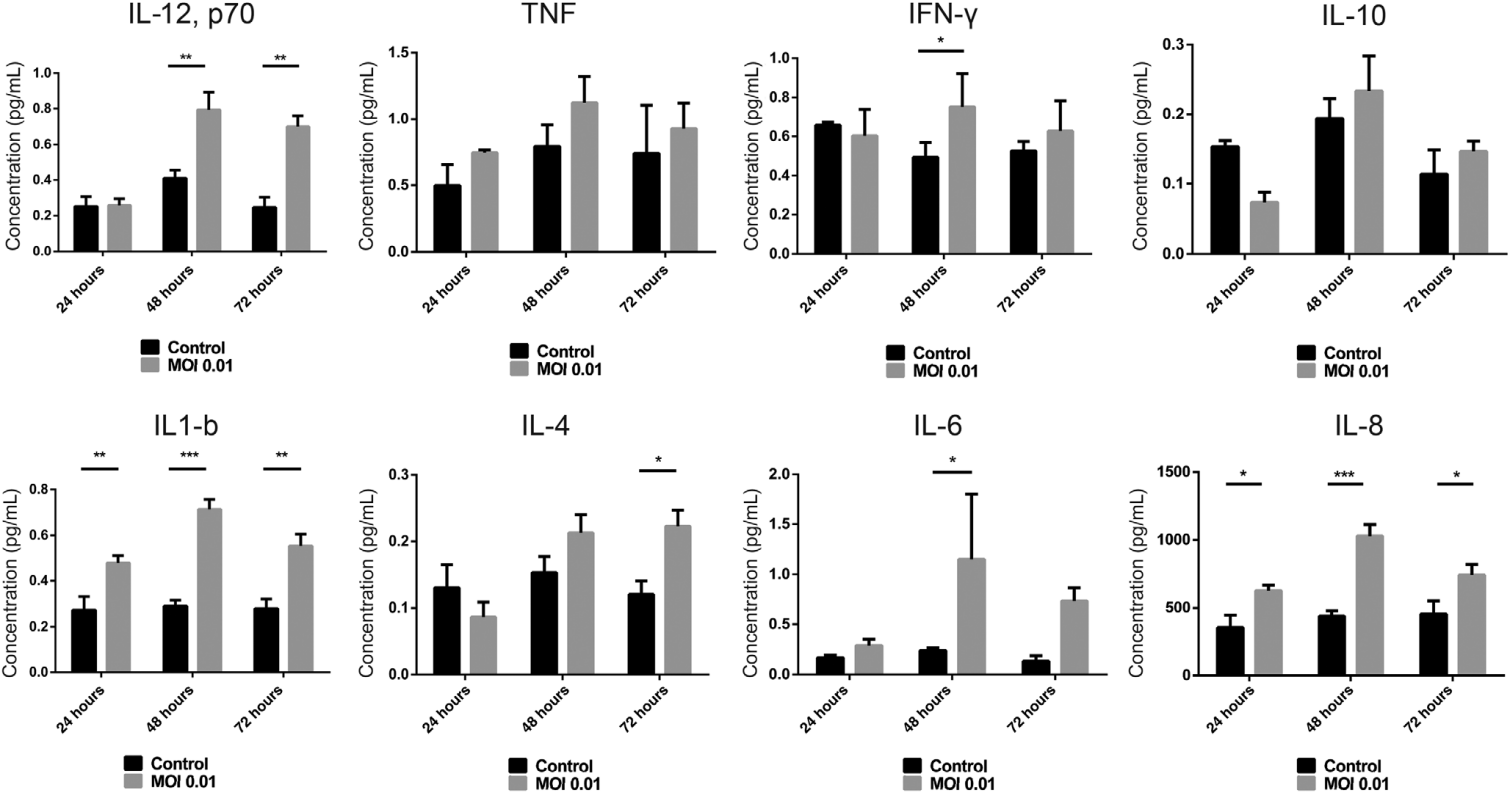
Lung airway constructs secrete inflammatory cytokines in response to SARS‐CoV‐2 infection. Data shown as mean ± SEM, n = 3

Airway constructs secrete inflammatory cytokines in response to SARS‐CoV‐2 infection (Figure 5). IL‐6, IL‐12, IL‐8, interferon‐gamma, and IL‐1β all show increased secretion 48 hours after infection; however, the greatest (and most statistically significant) increase is shown by IL‐6 equating to a fourfold increase above that of the uninfected control 48 hours postinfection. In patients with COVID‐19, IL‐6 levels are significantly elevated (>2.9‐fold greater than nondiseased individuals)^38^ and this is associated with adverse clinical outcomes. Increased cytokine secretion in the infected constructs was also observed for IL‐12, IL‐1β, IL‐4, and IL‐8 at 72 hours postinfection (Figure 5). Upregulation of cytokines as early as 36 hours postinfection has also been observed in a cellular model of the lung epithelium based on Calu‐3 cells and suggests the cell's response to SARS‐CoV‐2 infection, which may or may not be related to the release of SARS‐CoV2 virions into the supernatant.^39^ In support of this, it has been shown that SARS‐CoV‐2 induced IL‐1β secretion depends on the necroptosis, which is an immunogenic cell death that can trigger inflammatory responses through releasing inflammatory cytokine and cell damage‐associated molecular patterns.^39^

The plasma cytokine levels of COVID‐19 patients are not exclusively the product of infected epithelial cells as cytokines are also secreted by activated and/or infected immune cells.^39^ However, since the “damage” signals that initiate immune cell recruitment are mediated by cytokines secreted by epithelia, the ability of our model to reflect this characteristic supports its similarity to in vivo tissue. Secretion of IL‐1β is also a characteristic of COVID‐19 and since this cytokine is instrumental in the recruitment of monocytes, secretion by our model is a valuable characteristic.^40^ Similarly, increased plasma concentrations of IL‐8 and IL‐12 have been observed in COVID‐19 patients consistent with their roles in immune cell recruitment. Of potentially greater interest is the secretion of interferon‐γ, which is secreted at statistically higher levels in infected constructs vs control at 48 hours postinfection. Most respiratory viruses induce interferons in airway epithelial cells^41^ but IFN‐γ (interferon‐gamma) has been implicated in pathogenesis of SARS‐CoV‐2 infection in the human upper airway.^42^, ^43^

In conclusion, collectively our data show that airway epithelial constructs generated from iPSCs‐derived basal cells have promise as models of the response of the airway epithelia to viral infections.

## 4 DISCUSSION

In this study, we established an efficient method of isolation of iPSCs‐derived basal‐like cells from a mixed population of lung progenitors. Comparably with primary cells, when grown in BEGM and 3T3‐J2 feeders, the iPSC‐derived basal cells can self‐renew and form colonies. They express genes associated with a basal cell phenotype (KRT14, deltaNp63, NGFR, integrin alpha 6) while maintaining their multipotent capacity. One other group has reported the isolation of a basal cell‐like population from lung spheroids^44^ using a 3T3 feeder system with addition of Rho‐kinase inhibitor, but they only isolated a small number of cells which lost their basal cell characteristics. Our combination of the monlayer differentiation protocol and transfer the 3T3/Rho kinase inhibitor system at an early stage of development (differentiation day 14) provides an effective protocol for enriching airway basal‐like cells that can be maintained over multiple passages. During the preparation of this manuscript, data from another group were published describing the generation of airway basal‐like cells from iPSC^45^; however, this method relies upon FACS (Fluorescence Acrivated Cell Sorting) enrichment of NKX2.1+ lung progenitor cells followed by culture as lung organoids. Airway basal‐like cells are obtained by a second FACS enrichment step from the lung organoids; however, our protocol is much simpler to apply and generates basal‐like cells similar to those obtained by the published FACS enrichment‐based method.

The ability to produce substantial numbers of this cell type, which are sufficiently robust to recover well from cryopreservation, makes them applicable for the generation of larger numbers of airway constructs in a cost‐effective manner. The formation of a pseudostratified epithelium similar to that of the human upper airway by culture at an ALI recapitulates that observed for primary airway basal cells. The thickness of the epithelium is between five and eight layers with ciliated cells being present at the apical face albeit with fewer ciliated cells compared with epithelia derived from primary basal cells. Despite this, the ciliated cells are motile and beat within the same frequency range in both primary and iPSC‐derived constructs supporting their functional similarities. Moreover, video recordings support the presence of motile cilia on the apical surface of iPSC‐derived airway constructs (Video S1).

To evaluate the utility of the model for investigation of viral respiratory diseases, we infected iPSC‐derived ALI constructs with SARS‐CoV‐2. Our findings indicate the presence of surface proteins (ACE2 and TMPRSS2) to which the virus will attach and which are necessary for cell invasion. Viral replication occurred with release of virions at the apical surface of the constructs, which was corroborated by plaque‐forming unit assays and IF analyses.

In parallel to quantification of viral particles, secretion of cytokines, chemokines, and molecules which mount a defense against viral infection were analyzed, thus our model is probably a reasonable approximation of the human airway epithelium that may be useful for the investigation of respiratory infectious disease and the toxicity of diverse xenobiotic substances. The current model can be expanded to incorporate immune cell components such as monocytes, neutrophils, macrophages, and dendritic cells since monitoring the recruitment of such cells and their response to signals produced by the infected epithelial cells is important for understanding the consequences of viral infection. Inclusion of immune cell components will form the next stage of this work.

## CONFLICT OF INTEREST

The authors declared no potential conflicts of interest.

## AUTHOR CONTRIBUTIONS

I.D.: experimental design, data acquisition, data interpretation, and manuscript preparation; M.G.: data acquisition, data interpretation, and manuscript preparation; E.I.P., A.C.S., S.H.P., G.A.B.: data acquisition and interpretation; G.L.H., J.V.B.: experimental design and data acquisition; M.S.: experimental design; G.C.: data acquisition; M.L.: experimental design, funding, and manuscript preparation; L.A.: experimental design, data interpretation, funding, and manuscript preparation.

## Supporting information


**Figure S1** Analysis of iPSC‐derived airway basal‐like cells. A, Quantitative analysis of cytokeratin 14, integrin α‐6, and nerve growth factor receptor (NGFR) indicating enrichment of the iPSC‐derived basal‐like cell population generated from iPSCs (passage 4). B, Comparison of cytokeratin 14 and Integrin α‐6 expression by IF in iPSC‐derived and primary ex vivo lung airway basal cells

**Figure S2** Culture of iPSC‐derived basal cells on 3T3 mitotically inactivated feeders is necessary for their continued expansion. Representative bright field photos from iPSC‐derived basal cells are shown at the top panel. Those were expanded for two passages in the presence and absence of 3T3 mitotically inactivated feeder cells (bottom panel). The basal cells cultured in the absence of 3T3 mitotically inactivated feeder cells, could not be maintained past passage 4. All scale bars (red) represent 100 μm

**Figure S3** Quantitative real‐time PCR analysis showing expression of basal epithelial markers during 3T3‐J2 mediated expansion of iPSC‐derived basal epithelial cells. Data shown as mean ± SD, n = 3. Significance assessed by one‐way ANOVA. Ex vivo expanded basal epithelial cells from primary human lung tissue were used as calibrator.

**Figure S4** Confocal microscopy analyses of iPSC‐derived ALI constructs showing co‐expression of Spike, ACE2 and TMPRSS2 on the apical face in the SARS‐CoV‐2‐infected samples. *X*‐*Z* orthogonal reconstructions from *z*‐stacks covering the entire ALI construct within the *Z* axis; scale bars 50 μm. No Spike protein was detected in the mock infected controls.

**Video S1** Video recordings of motile cilia on the apical surface of iPSC‐derived airway constructs

## Data Availability

The data that support the findings of this study are available from the corresponding author upon reasonable request.
