# Supplementary figures and images for: Sars-Cov-2 Infects an Upper Airway Model Derived from Induced Pluripotent Stem Cells
Source: Stem Cells. 2021 Jun 21;39(10):1310–21. doi: 10.1002/stem.3422 (PMC8441770; doi:10.1002/stem.3422)

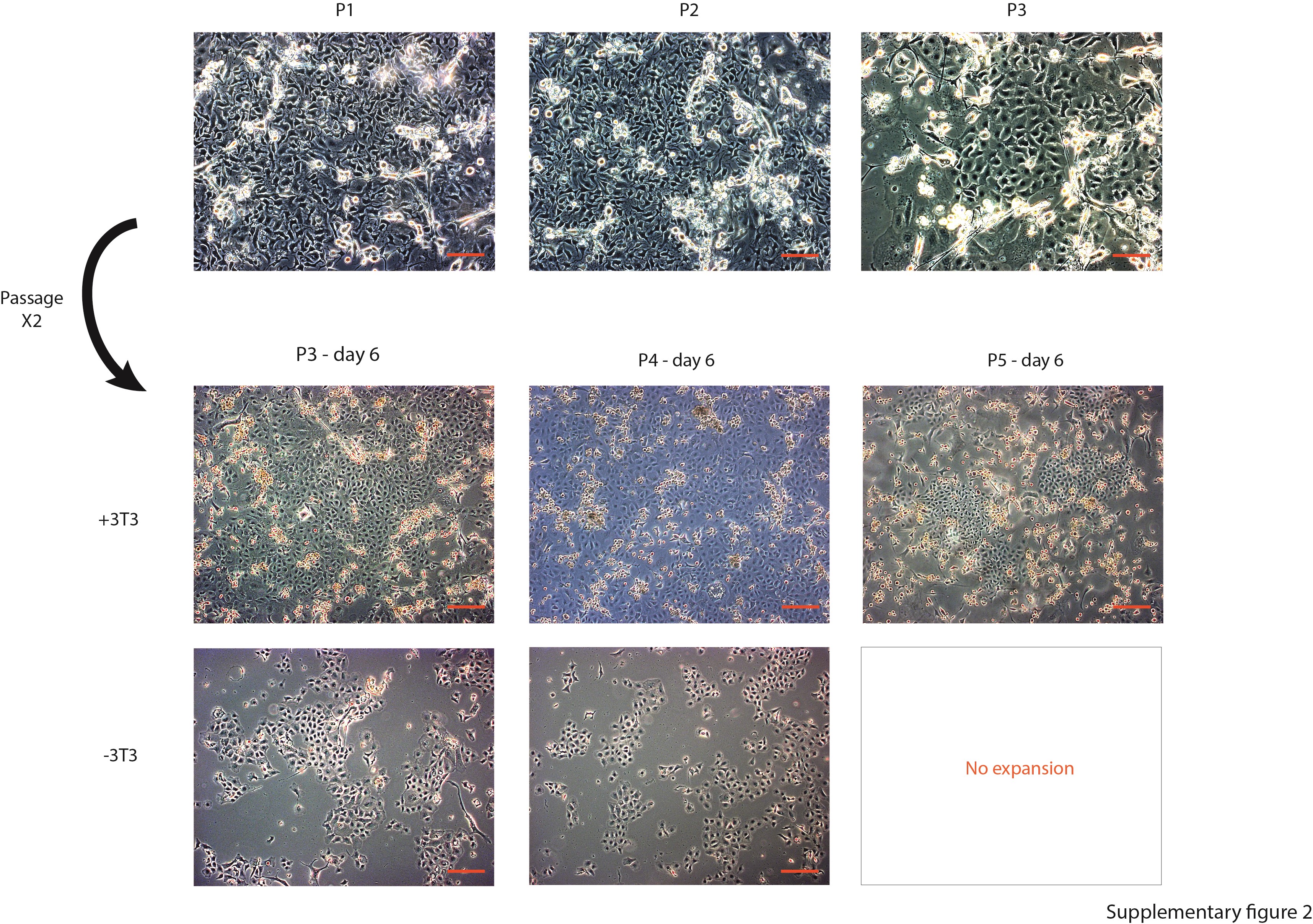

Supplement: stem3422-sup-0002-FigureS2 — Figure S2 Culture of iPSC-derived basal cells on 3T3 mitotically inactivated feeders is necessary for their continued expansion. Representative bright field photos from iPSC-derived basal cells are shown at the top panel. Those were expanded for two passages in the presence and absence of 3T3 mitotically inactivated feeder cells (bottom panel). The basal cells cultured in the absence of 3T3 mitotically inactivated feeder cells, could not be maintained past passage 4. All scale bars (red) represent 100 μm [file stem3422-sup-0002-figures2.jpeg]

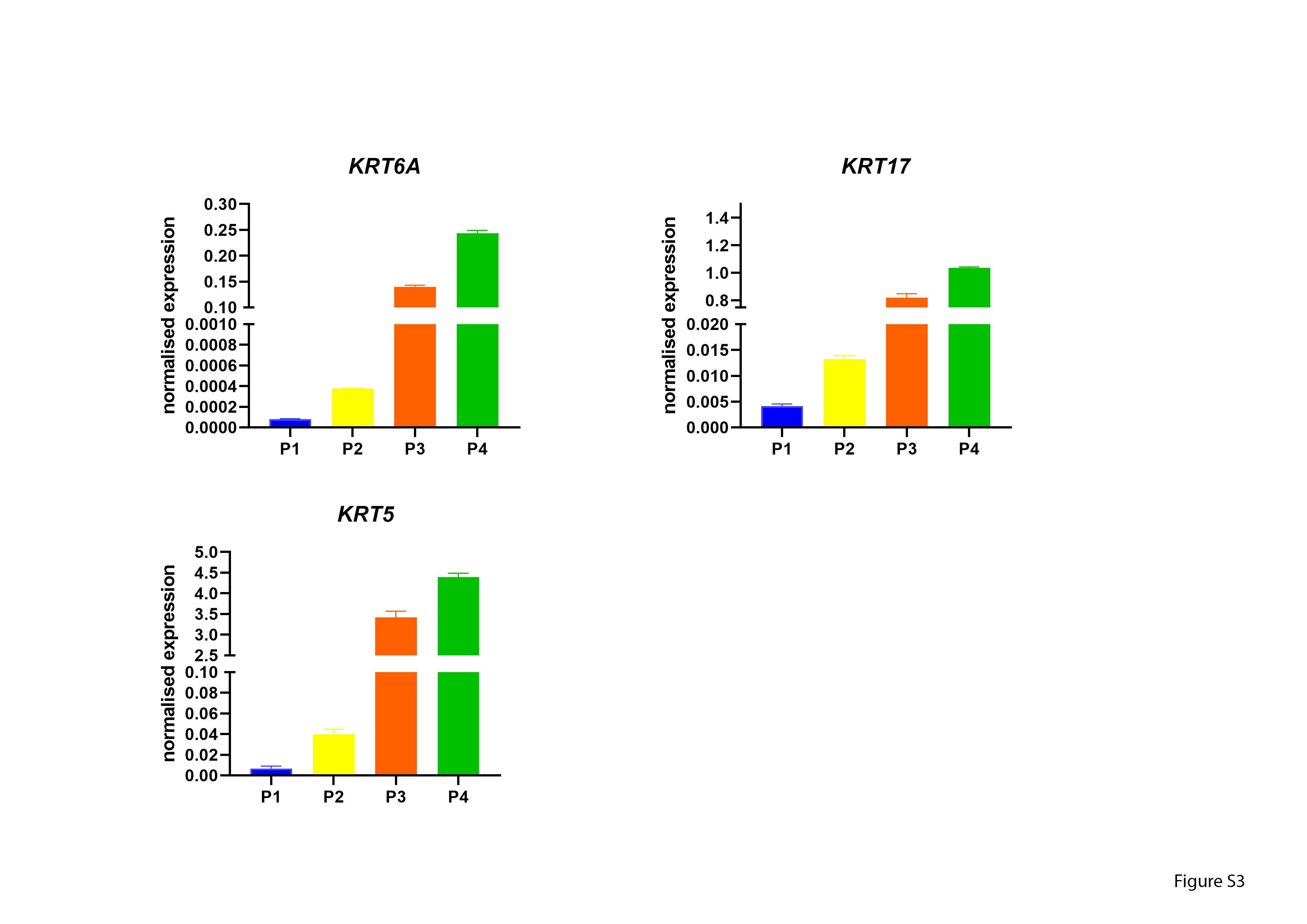

Supplement: stem3422-sup-0003-FigureS3 — Figure S3 Quantitative real-time PCR analysis showing expression of basal epithelial markers during 3T3-J2 mediated expansion of iPSC-derived basal epithelial cells. Data shown as mean ± SD, n = 3. Significance assessed by one-way ANOVA. Ex vivo expanded basal epithelial cells from primary human lung tissue were used as calibrator. [file stem3422-sup-0003-figures3.jpeg]

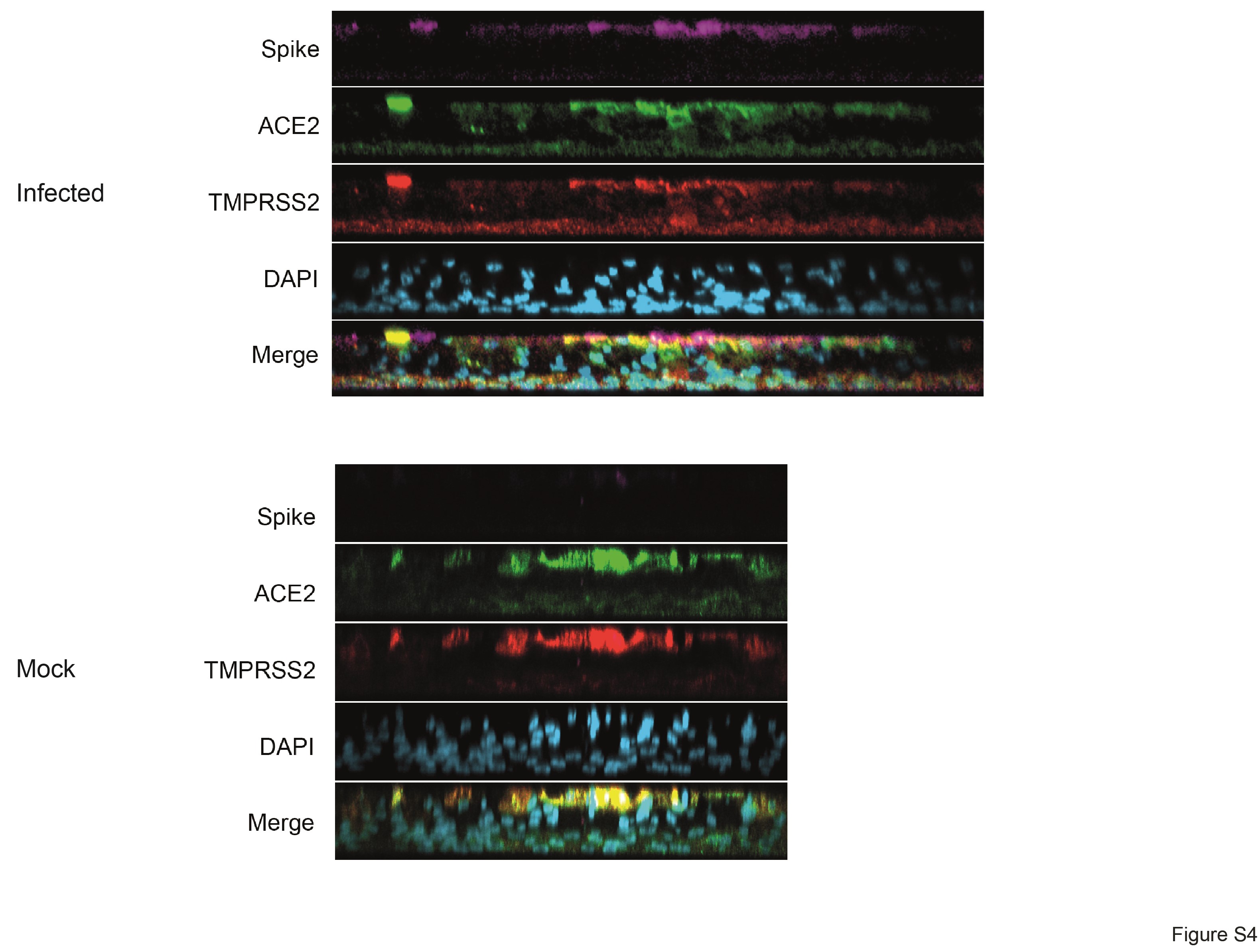

Supplement: stem3422-sup-0004-FigureS4 — Figure S4 Confocal microscopy analyses of iPSC-derived ALI constructs showing co-expression of Spike, ACE2 and TMPRSS2 on the apical face in the SARS-CoV-2-infected samples. X-Z orthogonal reconstructions from z-stacks covering the entire ALI construct within the Z axis; scale bars 50 μm. No Spike protein was detected in the mock infected controls. [file stem3422-sup-0004-figures4.jpeg]
